# Supplementary material for: Evidence of a Causal Association Between Cancer and Alzheimer’s Disease: a Mendelian Randomization Analysis
Source: Sci Rep. 2019 Sep 19;9:13548. doi: 10.1038/s41598-019-49795-6 (PMC6753207; doi:10.1038/s41598-019-49795-6)
Supplement: Supplementary file 1 — Supplementary File [file 41598_2019_49795_MOESM1_ESM.pdf]

Evidence of a Causal Association Between Cancer and Alzheimer's Disease: a Mendelian Randomization Analysis

Sahba Seddighi [1], Alexander L Houck [2], James B Rowe [3], Paul DP Pharoah [4*]

[1] University of Cambridge Institute of Public Health, University of Cambridge, Cambridge, UK

[2] University of Tennessee Health Science Center, Memphis, Tennessee, US

[3] Department of Clinical Neurosciences, University of Cambridge, Cambridge, UK

[4] Department of Oncology, Cambridge University Hospitals NHS Foundation Trust, Cambridge, UK

[*] Corresponding author (pp10001@medschl.cam.ac.uk)

Supplementary Table 1. Description of the International Genomics of Alzheimer's Project (IGAP) datasets. IGAP consists of four GWAS: The Cohort for Heart and Ageing Research in Genomic Epidemiology (CHARGE) consortium, the Alzheimer's Disease Genetics Consortium (ADGC), the Genetic and Environmental Risk in Alzheimer's Disease (GERAD) consortium, and the European Alzheimer's Disease Initiative (EADI) consortium. ADGC is the largest study, followed by GERAD, EADI, and CHARGE. Characteristics of controls and cases from each data collection site are provided below. N= number of participants; mean AAO= average age at AD-onset; Mean AAE= average age at study entry; SD= standard deviation.

Consortium	AD cases			Controls		
	N	% Female	Mean AAO (SD)	N	% Female	Mean AAE (SD)
**ADGC**						
ACT	566	63.1	83.9 (4.8)	1696	55.8	81.1 (6.0)
ADC	2512	52.6	73.4 (7.4)	1245	62.6	75.3 (8.6)
ADNI	268	42.2	73.0 (7.2)	173	40.5	78.6 (5.5)
GSK	669	56.8	74.6 (6.2)	713	64.0	73.8 (7.0)
NCRAD/NIA-LOAD	1811	64.9	73.6 (6.7)	1572	60.2	74.0 (8.5)
MAYO	728	57.6	-	1173	51.2	72.9 (4.3)
MIRAGE	509	63.7	71.2 (6.5)	742	58.1	72.0 (7.2)
OHSU	131	61.8	86.1 (5.5)	153	54.9	83.9 (7.6)
ROS/MAP	291	70.5	85.6 (6.3)	776	72.0	82.0 (7.0)
TGEN2	129	46.6	74.9 (7.2)	493	37.7	80.1 (8.7)
UM/VU/MSS	1070	63.7	73.9 (7.8)	1128	61.3	73.0 (7.1)
UPITT	1271	63.1	72.9 (6.4)	841	63.4	75.4 (6.1)
WASHU	318	55.7	74.2 (8.0)	187	60.4	76.9 (8.4)
**CHARGE**						

Consortium	AD cases			Controls		
	**N**	**% Female**	**Mean AAO (SD)**	**N**	**% Female**	**Mean AAE (SD)**
AGES	78	50.0	81.0 (5.0)	2694	58.0	76.0 (5.0)
CHS	421	65.8	81.1 (5.1)	1834	62.0	74.7 (4.6)
FHS	183	64.0	85.7 (7.2)	6300	49.4	73.0 (9.7)
Rotterdam Study	633	63.3	83 (8.0)	10938	59.0	69.0 (9.0)
**EADI**						
	2243	64.9	68.5 (8.9)	6017	60.7	74.0 (5.4)
**GERAD**						
	3177	64.0	73.0 (8.5)	7277	51.8	51.0 (11.8)

Supplementary Table 2. Alzheimer's disease clinical diagnostic criteria used in the International Genomics of Alzheimer's Project.[1-4] CHARGE = The Cohort for Heart and Ageing Research in Genomic Epidemiology consortium; ADGC= the Alzheimer's Disease Genetics Consortium; GERAD= the Genetic and Environmental Risk in Alzheimer's Disease consortium; EADI= the European Alzheimer's Disease Initiative consortium. NINCDS-ADRDA= National Institute of Neurological Disorders and Stroke-Alzheimer's Disease and Related Disorders Association criteria; MMSE= Mini-mental State Examination; DSM-IV= Diagnostic and Statistical Manual- Fourth Edition.

Consortium	Diagnostic criteria
ADGC	
ACT	NINCDS-ADRDA criteria for possible or probable AD
ADC	DSM-IV criteria or CDR ≧1 (70% autopsy-confirmed)
ADNI	NINCDS-ADRDA criteria for probable AD
GSK	NINCDS-ADRDA criteria and DSM-IV criteria for probable AD
NCRAD/NIA-LOAD	NINCDS-ADRD criteria for possible, probable, or definite AD
MAYO	NINCDS-ADRDA criteria for possible or probable AD (34% autopsy confirmed)
MIRAGE	NINCDS-ADRDA criteria for probable or definite AD
OHSU	Autopsy-confirmed AD
ROS/MAP	Clinical criteria for AD
TGEN2	Possible or probable AD (autopsy-confirmed)
UM/VU/MSS	NINCDS-ADRDA criteria for probable or definite AD (34% autopsy-confirmed)
UPITT	NINCDS-ADRDA criteria for probable or definite AD

Consortium	Diagnostic criteria
WASHU	Standard crieria
CHARGE	
AGES	NINCDS-ADRDA criteria for possible or probable AD
CHS	NINCDS-ADRDA criteria for possible or probable AD
FHS	NINCDS-ADRDA criteria for possible, probable, or definite AD
Rotterdam Study	NINCDS-ADRDA criteria for possible, probable, or definite AD
EADI	
	NINCDS-ADRDA criteria for probable AD
GERAD	
	NINCDS-ADRDA and DSM-IV criteria for probable AD and CERAD criteria for definite AD

Supplementary Table 3. Characteristics of studies included in literature review.

Reference	Study design	Study location	Mean follow-up (years)	Maximum sample size
Driver et al., 2012	Prospective cohort study	U.S.A	10	176 cancer patients; 1102 non-cancer controls
Musicco et al., 2013	Retrospective historical cohort study	Northern Italy	5	101,349 AD patients
Freedman et al., 2016	Prospective cohort study	U.S.A	Median: 1.9 (w/ cancer) 5.8 (w/out cancer)	742809 cancer patients; 420518 non-cancer controls
Frain et al., 2017	Retrospective cohort	U.S.A	Median: 5.7	2,728,093 cancer patients; 771,285 non-cancer controls
Schmidt et al., 2017	Prospective cohort study	Denmark	Median: 5.8 cancer; 5.6 non-cancer	216,221 cancer patients; 1,081,097 non-cancer controls
Chung et al., 2016	Retrospective cohort study	Taiwan	5	1335 cancer patients; 4005 non-cancer controls
White et al., 2013	Prospective cohort study	New York City, USA	3.7	141 cancer patients; 961 non-cancer controls
Realmuto et al., 2012	Case-control study	Palermo, Italy	-	126 AD patients; 252 non-AD patients
Bowles et al., 2017	Prospective cohort study	Washington State, USA	Median: 6.4	1339 cancer patients; 3018 non-cancer controls
Yarchoan et al., 2017	Prospective cohort study	U.S.A.	6.7 (w/ cancer); 6.9 (w/out cancer)	401 cancer patients; 888 non-cancer controls
Roe et al., 2010	Prospective cohort study	Forsyth County, NC; Washington County, MD; Sacramento County, CA; Pittsburgh, PA	5.4	898 cancer patients; 2122 non-cancer controls

Supplementary Table 4. Characteristics of studies included in literature review (continued).

Reference	Cancer case ascertainment	AD diagnostic criteria	Comments
Driver et al., 2012	ICD-O code	NINCDS- ADRDA criteria	Strength: long follow-up period. Limitations: did not include those who died of cancer before study, who may be at different risk than survivors; participants were mostly white, so limited generalizability; could not assess effect of cancer treatment
Musicco et al., 2013	ICD-10 code	Based on registries of drug prescriptions, hospitalizations, and payment exemptions	Limitation of using registry data: could not examine role of risk factors for cancer or AD, particularly lifestyle factors; case ascertainment was based on administrative data, which might lack sensitivity, hurting validity of their study; AD was ascertained based on drug prescriptions, hospitalization, and payment exemptions, so many cases could have been missed; cancer was ascertained through hospital discharge forms, but not all cancer patients require hospitalization.
Freedman et al., 2016	ICD-O-3 code	ICD-9 code	Limitations: used Medicare data, which relies on claims, rather than validated clinical diagnoses, and this could lead to underascertainment and misclassification of AD, as well as incorrect assignment of diagnosis date; misclassification can bias results towards null; restricted to Medicare eligible individuals; could not account for treatment effects, which may confound the relationship between cancer and AD.
Frain et al., 2017	ICD-9 code	ICD-9 code	Limitations: used retrospective chart analysis to eliminate prevalent AD at baseline, but there is a threat of severe underdiagnosis; using diagnostic codes for case ascertainment can lead to substantial misclassification; did not have information on cancers that were diagnosed and treated outside of the VA.
Schmidt et al., 2017	ICD-O-3 code	ICD-8 & ICD-10 codes	Limitations: could not account for lifestyle risk factors for AD; used retrospective data for AD and cancer diagnoses
Chung et al., 2016	ICD-9 code	ICD-9-CM code	Limitations: short follow-up period might not allow the sampled patients to develop AD; population was mostly of Han Chinese ethnicity, so limited generalizability; could not account for risk factors of AD.
White et al., 2013	Semi-structured questionnaire	DSM-IV & NINDCDS ARDA	Limitations: cancer diagnosis based on self-report; strengths: uses well-established procedures to ascertain AD.

Reference	Cancer case ascertainment	AD diagnostic criteria	Comments
Realmuto et al., 2012	Semi-structured questionnaire to AD caregivers or controls	DSM-IV & NINDCDS ARDA	Limitation: cancer diagnosis in AD patients was based on medical records, but no information from care givers to supplement
Bowles et al., 2017	semi-structured questionnaire	DSM-IV & NINDCDS ARDA	Limitations: could not examine effect of cancer treatment on risk of AD; relatively new registry, so prior cancer diagnosis could be missed; mostly white individuals, limiting generalizability.
Yarchoan et al., 2017	Semi-structured questionnaire	NINCDS-ADRDA criteria	Strength: High follow-up rate (>90%) and autopsy rate (>86%)l; corroboration with pathological markers of AD. Limitations: self-reported cancer history; could not examine effect of cancer treatment on relationship; could not determine if those who died from cancer at a younger age would have similar risk of AD as the cancer survivors; mostly white participants, so limited generalizability.
Roe et al., 2010	ICD-9 code	NINCDS-ADRDA criteria	Cancers treated on an outpatient basis were not included; only included cancer survivors, not those who died of cancer

Supplementary Table 5. Total number of SNPs identified as instrumental variables for each cancer type in European populations. Existing genome-wide associated studies from the GWAS Catalog (https://www.ebi.ac.uk/gwas/) were used to identify cancer-associated genetic variants at genome-wide significance ($p<5.00E-8$). Only studies of European populations were included, and the lead SNP at each locus was selected. Finally, cancer-associated loci were checked for linkage disequilibrium ($r^2>0.8$), in which case the SNP with the lowest p-value was selected, yielding the final list of SNPs below.

Cancer type	Study	N controls	N cases	N lead SNPs retrieved for MR
Renal cell carcinoma				
	Henrion et al., 2015	10755	2598	1
	Henrion et al., 2013	37352	5954	1
	Wu et al., 2012	10021	4666	1
	Purdue et al., 2010	13423	5970	2
	Scelo et al., 2017	34038	19164	7
Pancreatic cancer				
	Zhang et al., 2016	16400	11183	3
	Peterson et al., 2010	3934	3851	2
	Wolpin et al., 2014	14397	7683	5

Cancer type	Study	N controls	N cases	N lead SNPs retrieved for MR
	Childs et al., 2015	11569	4164	3
Upper aerodigestive tract cancer				
	Lesseur et al., 2017	6585	6034	11
	McKay et al., 2011	18199	13697	3
Urinary bladder cancer				
	Figueroa et al., 2014	18872	10134	2
	Rafnar et al., 2011	11329	6024	1
	Rothman et al., 2010	53395	11913	4
	Wu et al., 2009	40547	7636	1
	Kiemenet et al., 2008	38136	3968	1
	Keimeney et al., 2010	45549	4739	1
	Rafnar et al., 2014	100636	6936	1
Lung cancer				
	McKay et al., 2017	68712	44069	16
	Wang et al., 2014	38295	21594	2
Cervical cancer				
	Chen et al., 2016	6167	2503	1
Prostate cancer				
	Knipe et al., 2014	3241	3000	4
	Eeles et al., 2013	24272	25074	21
	Gudmundsson et al., 2012	54444	4537	2
	Gudmundsson et al., 2009	42382	3949	3
	Gudmundsson et al., 2008	21372	1854	2
	Eeles et al., 2008	5260	5122	3
	Gudmundsson et al., 2007	11290	1,501	1
Leukemia				
	Ellinghaus et al., 2012	3535	1370	4

Cancer type	Study	N controls	N cases	N lead SNPs retrieved for MR
	Papaemmanuil et al., 2009	2398	907	2
	Sherborne et al., 2010	4817	3,293	1
	Law et al., 2017	17598	6200	9
	Berndt et al., 2016	13197	5058	3
	Berndt et al., 2013	10521	5443	9
	Slager et al., 2012	5778	2072	1
	Bernardo et al., 2008	3115	1529	7
	Crowther-Swanpoel et al., 2010	5789	2503	2
Breast cancer				
	Michailidou et al., 2017	105974	122977	108
	Milne et al., 2017	100594	21468	1
Melanoma				
	Law et al., 2015	26409	15990	7
	Ransohoff et al., 2017	287591	6628	6
	Iles et al., 2013	59233	13666	1
	Barrett et al., 2011	8408	2981	3
	MacGregor et al., 2011	19531	7361	2
	Bishop et al., 2009	6203	3962	4
	Brown et al., 2008	2105	2019	1
Lymphoma				
	Cerhan et al., 2014	12223	5216	5
	Sud et al., 2017	16749	5314	6
	Cozen et al., 2014	11095	3097	1
	Frampton et al., 2013	8441	3489	1
	Enciso-Mora et al., 2010	8615	2646	2
	Vijai et al., 2013	6640	2189	1
	Skibola et al., 2014	13344	4523	4

Cancer type	Study	N controls	N cases	N lead SNPs retrieved for MR
Ovarian cancer				
	Bolton et al., 2010	13184	10507	1
	Goode et al., 2010	13185	10283	3
	Song et al., 2009	11840	8761	1
	Kelemen et al., 2015	21693	1644	2
	Phelan et al., 2017	40941	25509	8
	Kuchenbaecker et al., 2015	30845	18533	1
	Pharoah et al., 2013	26134	18174	3

Supplementary Table 6. Summary statistics for genetic variants used in Mendelian randomization. *Retrieved from NCBI dbSNP tool; SNP= single nucleotide polymorphism; EA= effect allele; RA= reference (non-effect) allele; Beta= beta-coefficient; SE= standard error.

Cancer type	SNP	Locus**	Nearest gene*	EA	RA	Beta	SE	p-value	Beta	SE	p-value
							Exposure			Outcome	
Renal cell carcinoma											
	rs10936602	3q26.2	*LRRIQ4*	C	T	-0.105	0.018	0.000	0.026	0.019	0.168
	rs11813268	10q24.33-q25.1	-	T	C	0.113	0.021	0.000	-0.053	0.022	0.015
	rs11894252	2p21	*EPAS1*	T	C	0.148	0.019	0.000	0.010	0.016	0.540
	rs12105918	2q22.3	*ZEB2*	C	T	0.223	0.038	0.000	0.044	0.034	0.191
	rs2241261	8p21.3	*RHOBTB2 - LOC107986927*	T	C	0.095	0.016	0.000	-0.005	0.016	0.770
	rs3845536	1q24.1	*ALDH9A1*	C	T	0.191	0.034	0.000	-0.018	0.017	0.284
	rs4381241	1p32.3	*FAF1*	C	T	0.104	0.017	0.000	-0.005	0.016	0.763
	rs4765623	12q24 .31	*SCARB1*	T	C	0.131	0.019	0.000	-0.040	0.017	0.019
	rs4903064	14q24.2	*DPF3*	C	T	0.191	0.019	0.000	-0.003	0.019	0.888
	rs67311347	3p22.1	-	A	G	-0.105	0.019	0.000	-0.029	0.019	0.130
	rs7105934	11q13.3	*LOC102724265*	A	G	-0.357	0.037	0.000	-0.019	0.028	0.498

Cancer type	SNP	Locus**	Nearest gene*	EA	RA	Beta	SE	p-value	Beta	SE	p-value
							Exposure			Outcome	
	rs718314	12p11.23	*LOC105369705*	C	T	0.166	0.020	0.000	-0.001	0.018	0.943
	rs74911261	11q22.3	*KDELC2*	A	G	0.344	0.054	0.000	-0.079	0.085	0.350
Pancreatic cancer											
	rs10094872	8q24.21	*CASC11*	T	A	0.140	0.024	0.000	-0.040	0.018	0.029
	rs11655237	17q25.1	*LINC00673*	T	C	0.231	0.030	0.000	-0.042	0.026	0.100
	rs16986825	22q12.1	*ZNRF3*	T	C	0.166	0.029	0.000	-0.013	0.022	0.542
	rs17688601	7p13	*SUGCT*	A	C	-0.128	0.023	0.000	0.033	0.017	0.060
	rs2736098	5p15.33	*TERT*	T	C	-0.223	0.030	0.000	-0.044	0.024	0.070
	rs2816938	1q32.1	-	A	T	0.182	0.023	0.000	-0.013	0.019	0.498
	rs3790844	1q32.1	*NR5A2*	C	T	-0.261	0.041	0.000	0.000	0.018	0.985
	rs6971499	7q32.3	*LINC-PINT*	C	T	-0.236	0.034	0.000	0.003	0.026	0.897
	rs7190458	16q23.1	*BCAR1*	A	G	0.378	0.059	0.000	0.034	0.047	0.473
	rs9543325	13q22.1	-	C	T	0.231	0.035	0.000	0.010	0.016	0.555
	rs9581943	13q12.2	*PDX1 - PLUT*	A	G	0.140	0.023	0.000	-0.008	0.016	0.629
	rs9854771	3q29	*TP63*	A	G	-0.117	0.021	0.000	-0.001	0.016	0.931
Upper aerodigestive tract cancer											
	rs10462706	5p15.33	*CLPTM1L*	T	C	-0.301	0.049	0.000	-0.031	0.030	0.306
	rs1229984	4q23	*ADH1B*	T	C	-0.446	0.048	0.000	0.044	0.042	0.295
	rs1494961	4q21	*HELQ*	C	T	0.113	0.020	0.000	-0.015	0.016	0.357
	rs4767364	12q24	*NAA25*	A	G	0.122	0.022	0.000	-0.016	0.017	0.354
	rs6547741	2p23.3	*GPN1*	A	G	-0.186	0.034	0.000	-0.009	0.015	0.541
	rs8181047	9p21.3	*CDKN2B-AS1*	A	G	0.215	0.037	0.000	-0.035	0.017	0.040
	rs9274626	6p21	*HLA-DQB1*	C	T	0.247	0.034	0.000	-0.007	0.022	0.755
	rs928674	9q34.12	*LAMC3*	G	A	0.285	0.051	0.000	0.001	0.025	0.975
Urinary bladder cancer											
	rs10936599	3q26.2	*MYNN*	T	C	-0.163	0.028	0.000	0.014	0.018	0.433
	rs17674580	18q12.3	*LOC105372093 - SLC14A1*	T	C	0.157	0.024	0.000	-0.043	0.016	0.008

Cancer type	SNP	Locus**	Nearest gene*	EA	RA	Beta	SE	p-value	Beta	SE	p-value
							Exposure			Outcome	
	rs2294008	8q24.3	*PSCA*	T	C	0.122	0.019	0.000	0.004	0.016	0.808
	rs62185668	20p12.2	*C20orf187*	A	C	0.174	0.026	0.000	-0.017	0.018	0.342
	rs710521	3q28	-	A	G	0.166	0.026	0.000	0.054	0.017	0.002
	rs798766	4p16.3	*TACC3*	T	C	0.182	0.025	0.000	-0.014	0.020	0.494
	rs907611	11p15.5	*LSP1*	A	G	0.140	0.025	0.000	-0.025	0.018	0.164
	rs9642880	8q24.21	*CASC11*	T	G	0.191	0.022	0.000	-0.021	0.016	0.197
Lung cancer											
	rs1056562	11q23.3	*MPZL2*	T	C	0.095	0.015	0.000	-0.012	0.017	0.472
	rs11591710	10q24.3	-	C	A	0.182	0.028	0.000	-0.057	0.023	0.013
	rs116822326	6p21.33	-	G	A	0.262	0.029	0.000	-0.011	0.023	0.644
	rs11780471	8p21.1	-	A	G	-0.105	0.019	0.000	0.032	0.032	0.306
	rs13080835	3q28	*TP63*	T	G	-0.105	0.015	0.000	-0.002	0.016	0.878
	rs146782107	chr15:49366480	-	C	T	-0.105	0.013	0.000	0.003	0.018	0.880
	rs41309931	20q13.33	*RTEL1-TNFRSF6B - RTEL1 - TNFRSF6B*	T	G	0.182	0.030	0.000	0.010	0.029	0.735
	rs4236709	8p12	*NRG1*	G	A	0.095	0.015	0.000	-0.004	0.019	0.815
	rs55781567	15q25.1	*CHRNA5*	G	C	0.262	0.012	0.000	-0.046	0.017	0.006
	rs56113850	19q13.2	*CYP2A6*	T	C	-0.105	0.012	0.000	-0.027	0.023	0.235
	rs6920364	6q27	-	C	G	0.095	0.017	0.000	0.014	0.016	0.397
	rs71658797	1p31.1	*AK5*	A	T	0.095	0.014	0.000	-0.037	0.028	0.182
	rs7705526	5p15.33	*TERT*	A	C	0.262	0.021	0.000	-0.063	0.024	0.008
	rs7953330	12p13.33	*WNK1*	C	G	-0.105	0.015	0.000	-0.008	0.018	0.669
	rs885518	9p21.3	*MTAP*	G	A	0.182	0.030	0.000	0.032	0.025	0.192
Cervical cancer											
	rs9272005	6p21.3	*LOC107987449 - LOC107987459*	G	A	-0.446	0.044	0.000	0.038	0.023	0.089
Prostate cancer											
	rs1056441	chr:20,62354144	*LIME1 - SLC2A4RG*	T	C	-0.117	0.014	0.000	0.016	0.018	0.370

Cancer type	SNP	Locus**	Nearest gene*	EA	RA	Beta	SE	p-value	Beta	SE	p-value
							**Exposure**			**Outcome**	
	rs10934853	rs10934853	*EEFSEC*	A	C	0.113	0.018	0.000	0.006	0.017	0.733
	rs10993994	10q11	*MSMB*	T	C	0.400	0.038	0.000	0.005	0.017	0.765
	rs11135910	chr8:25948059	*EBF2*	A	G	0.104	0.016	0.000	-0.016	0.022	0.463
	rs11228565	11q13	-	A	G	0.207	0.030	0.000	-0.017	0.019	0.377
	rs11568818	11q22.2	*MMP7*	G	A	-0.094	0.014	0.000	-0.034	0.016	0.034
	rs11902236	chr2: 10035319	*GRHL1*	A	G	0.068	0.012	0.000	0.014	0.018	0.452
	rs12155172	chr7: 20961016	*LINC01162*	A	G	0.104	0.014	0.000	-0.001	0.020	0.957
	rs1218582	chr1: 153100807	*KCNN3*	G	A	0.058	0.010	0.000	0.004	0.016	0.796
	rs1270884	chr12:113169954	-	A	G	0.068	0.010	0.000	-0.030	0.016	0.059
	rs1447295	8q24	*CASC8*	A	C	0.510	0.059	0.000	-0.065	0.026	0.013
	rs17632542	19q13.33	*KLK3*	T	C	0.730	0.066	0.000	-0.012	0.031	0.699
	rs1894292	chr4:74568022	*AFM*	A	G	-0.094	0.013	0.000	0.003	0.016	0.849
	rs1933488	chr6:153482772	*RGS17*	G	A	-0.117	0.013	0.000	0.005	0.016	0.753
	rs2273669	chr6:109391882	*ARMC2*	G	A	0.068	0.012	0.000	0.005	0.021	0.824
	rs2427345	chr20:60449006	*LOC105372710*	A	G	-0.062	0.011	0.000	0.019	0.018	0.288
	rs2660753	3p12.1	-	T	C	0.419	0.078	0.000	0.019	0.025	0.450
	rs266849	19q13	*LOC105372441*	G	A	-0.478	0.058	0.000	0.024	0.020	0.226
	rs3771570	chr2:242031537	*FARP2*	A	G	0.113	0.019	0.000	0.007	0.024	0.759
	rs3850699	chr10:104404211	*LOC105378460 - TRIM8*	G	A	-0.094	0.015	0.000	0.016	0.017	0.337
	rs4245739	1q32	*MDM4*	C	A	-0.094	0.014	0.000	0.034	0.018	0.056
	rs4430796	17q12	*HNF1B*	A	G	0.199	0.029	0.000	-0.006	0.017	0.739
	rs4793529	17q24	*CASC17*	T	C	0.280	0.038	0.000	0.020	0.016	0.228
	rs6465657	7q21	*LMTK2*	C	T	0.262	0.046	0.000	0.003	0.016	0.846
	rs684232	chr17:565715	*VPS53*	G	A	0.095	0.012	0.000	0.031	0.017	0.065
	rs6869841	chr5:172872032	*LOC105377732*	A	G	0.068	0.012	0.000	-0.018	0.019	0.337
	rs7141529	14q24	*RAD51B*	G	A	0.086	0.014	0.000	-0.005	0.016	0.778

Cancer type	SNP	Locus**	Nearest gene*	EA	RA	Beta	SE	p-value	Beta	SE	p-value
							Exposure			Outcome	
	rs721048	2p15	*EHBP1*	A	G	0.140	0.024	0.000	-0.030	0.021	0.146
	rs7241993	chr18:74874961	*LOC105372225*	A	G	-0.083	0.014	0.000	0.013	0.021	0.519
	rs7611694	chr3:114758314	*SIDT1*	C	A	-0.094	0.013	0.000	0.019	0.016	0.235
	rs8008270	chr14:52442080	*FERMT2 - LOC105370500*	A	G	-0.117	0.015	0.000	-0.012	0.020	0.564
	rs8102476	19q13.2	-	C	T	0.113	0.017	0.000	0.019	0.016	0.237
Leukemia											
	rs1036935	18q21.1	-	A	G	0.140	0.025	0.000	-0.019	0.019	0.303
	rs10936599	3q26.2	*MYNN*	C	T	0.231	0.038	0.000	-0.014	0.018	0.433
	rs11083846	19q13.32	*PRKD2*	A	G	0.300	0.051	0.000	0.022	0.019	0.238
	rs13397985	2q37.1	*SP110 - SP140*	G	T	0.344	0.055	0.000	0.011	0.021	0.601
	rs13401811	2q13	*ACOXL*	G	A	0.344	0.039	0.000	0.010	0.020	0.630
	rs1353286	6p21.3	-	G	T	0.174	0.026	0.000	-0.003	0.016	0.843
	rs140522	22q13.33	*ODF3B*	T	C	0.140	0.023	0.000	-0.027	0.019	0.145
	rs17200824	4q10	*BANK1*	A	G	0.157	0.024	0.000	-0.013	0.017	0.460
	rs17246404	7q31.33	*POT1*	C	T	0.199	0.036	0.000	-0.001	0.018	0.967
	rs17483466	2q13	*ACOXL*	G	A	0.329	0.052	0.000	-0.015	0.019	0.422
	rs17505102	3q28	*TP63*	C	G	-0.431	0.075	0.000	0.017	0.025	0.501
	rs1945213	11q11	-	C	G	-0.371	0.057	0.000	-0.002	0.018	0.898
	rs210142	6p21.33	*BAK1*	C	T	0.336	0.042	0.000	-0.025	0.017	0.156
	rs2236256	6q25.2	*IPCEF1 - OPRM1*	C	A	0.207	0.032	0.000	-0.010	0.016	0.502
	rs2239633	14q11.2	*CEBPE*	T	C	-0.301	0.048	0.000	0.037	0.016	0.023
	rs34676223	1p36.11	-	C	A	0.174	0.024	0.000	-0.016	0.018	0.373
	rs3731217	9p21.3	*CDKN2A*	G	T	-0.342	0.052	0.000	-0.013	0.023	0.553
	rs3769825	2q33.1	*CASP8*	T	C	0.174	0.029	0.000	-0.002	0.016	0.900
	rs3770745	2p22.2	*QPCT*	T	C	0.215	0.038	0.000	0.027	0.021	0.193
	rs3800461	6p21.31	*C6orf106*	C	G	0.182	0.032	0.000	-0.040	0.032	0.207
	rs41271473	1q42.13	*RHOU*	G	A	0.174	0.027	0.000	-0.022	0.019	0.257

Cancer type	SNP	Locus**	Nearest gene*	EA	RA	Beta	SE	p-value	Beta	SE	p-value
							Exposure			Outcome	
	rs4132601	7p12.2	*IKZF1*	C	A	0.525	0.058	0.000	0.013	0.018	0.464
	rs4368253	18q21.32	-	C	T	0.174	0.031	0.000	0.005	0.017	0.779
	rs4406737	10q23.31	*FAS*	G	A	0.239	0.031	0.000	-0.007	0.015	0.664
	rs4987855	18q21.33	*BCL2*	G	A	0.385	0.055	0.000	-0.022	0.027	0.405
	rs57214277	4q35.1	-	T	C	0.122	0.022	0.000	0.012	0.017	0.487
	rs61904987	11q23.2	*LOC107984390*	T	C	0.215	0.032	0.000	0.019	0.025	0.432
	rs6858698	4q26	*CAMK2D*	C	G	0.270	0.046	0.000	-0.009	0.027	0.754
	rs7089424	10q21.2	*ARID5B*	C	A	0.501	0.056	0.000	-0.022	0.017	0.186
	rs7176508	15q23	-	A	G	0.315	0.045	0.000	-0.018	0.016	0.258
	rs7254272	19p13.3	-	A	G	0.157	0.029	0.000	0.006	0.021	0.796
	rs735665	11q24.1	*GRAMD1B*	A	G	0.372	0.053	0.000	-0.047	0.020	0.017
	rs73718779	6p25.2	*SERPINB6*	T	C	0.231	0.041	0.000	-0.026	0.028	0.349
	rs757978	2q37.3	*FARP2*	A	G	0.329	0.055	0.000	-0.036	0.027	0.195
	rs7944004	11p15.5	-	T	G	0.182	0.029	0.000	-0.014	0.017	0.422
	rs8024033	15q15.1	-	C	G	0.199	0.031	0.000	-0.031	0.018	0.084
	rs872071	6p25.3	*IRF4*	G	A	0.432	0.047	0.000	-0.011	0.016	0.463
	rs897586	6p21.3	-	A	G	0.231	0.038	0.000	0.003	0.017	0.840
	rs898518	4q25	*LEF1*	A	C	0.182	0.029	0.000	0.007	0.016	0.673
	rs920590	8p21.3	-	C	T	0.307	0.053	0.000	0.012	0.017	0.462
	rs9308731	2q13	*BCL2L11*	A	G	0.174	0.026	0.000	0.013	0.016	0.431
	rs9378805	6p21.3	-	C	A	0.412	0.046	0.000	-0.006	0.015	0.695
Breast cancer											
	rs10022462	4q22.1	*LOC105369192*	T	C	0.037	0.006	0.000	0.006	0.016	0.712
	rs1011970	9p21.3	*CDKN2B-AS1*	T	G	0.066	0.008	0.000	0.028	0.021	0.191
	rs10474352	5q14.3	-	T	C	-0.059	0.009	0.000	0.025	0.022	0.254
	rs1053338	3p14.1	*ATXN7*	G	A	0.059	0.009	0.000	-0.004	0.023	0.866
	rs10760444	9q33.3	*LMX1B*	A	G	-0.036	0.006	0.000	0.027	0.016	0.089

Cancer type	SNP	Locus**	Nearest gene*	EA	RA	Beta	SE	p-value	Beta	SE	p-value
							Exposure			Outcome	
	rs10941679	5p12	-	G	A	0.128	0.007	0.000	0.007	0.021	0.747
	rs10995201	10q21.2	*ZNF365*	G	A	-0.132	0.009	0.000	-0.030	0.022	0.176
	rs11117758	1q41	*ESRRG*	A	G	-0.045	0.008	0.000	-0.021	0.019	0.272
	rs11199914	10q26.12	-	T	C	-0.046	0.007	0.000	0.001	0.018	0.947
	rs11249433	1p11.2	*EMBP1*	G	A	0.099	0.006	0.000	-0.013	0.016	0.426
	rs113577745	2p25.1	*GRHL1*	G	C	0.064	0.010	0.000	0.000	0.028	0.999
	rs11552449	1p13.2	*AP4B1 - DCLRE1B*	T	C	0.054	0.008	0.000	-0.015	0.024	0.520
	rs11627032	14q32.12	*RIN3*	C	T	-0.048	0.007	0.000	0.007	0.018	0.683
	rs117618124	18q12.1	*GAREM1*	C	T	-0.107	0.016	0.000	0.022	0.039	0.569
	rs11820646	11q24.3	-	C	T	0.048	0.006	0.000	-0.006	0.016	0.716
	rs11977670	7q34	*LOC107986854*	A	G	0.052	0.006	0.000	0.011	0.016	0.484
	rs12405132	1q21.1	*RNF115*	T	C	-0.041	0.007	0.000	-0.023	0.017	0.165
	rs12479355	2q36.3	-	G	A	-0.043	0.008	0.000	0.011	0.019	0.580
	rs12710696	2p24.1	-	C	T	-0.036	0.006	0.000	-0.004	0.016	0.812
	rs13267382	8q23.3	*LINC00536*	G	A	-0.044	0.006	0.000	-0.020	0.019	0.295
	rs13281615	8q24.21	*CASC21 - CASC8*	G	A	0.100	0.006	0.000	0.020	0.016	0.198
	rs13329835	16q23.2	*CDYL2*	G	A	0.079	0.007	0.000	-0.019	0.018	0.304
	rs13365225	8p11.23	-	G	A	-0.077	0.008	0.000	0.042	0.021	0.044
	rs1432679	5q33.3	*EBF1*	T	C	-0.072	0.006	0.000	-0.005	0.016	0.755
	rs16991615	20p12.3	*MCM8*	A	G	0.076	0.013	0.000	-0.013	0.032	0.693
	rs1707302	1p34.1	*LOC101929626 - LOC110117498-PIK3R3 - PIK3R3*	G	A	0.036	0.007	0.000	0.003	0.017	0.878
	rs17156577	7p15.1	*CREB5*	C	T	0.058	0.010	0.000	-0.006	0.028	0.833
	rs17268829	7q21.3	*LOC105375404*	C	T	0.049	0.007	0.000	0.010	0.017	0.545
	rs17426269	1p22.3	-	A	G	0.049	0.009	0.000	0.008	0.021	0.697
	rs17529111	6q14.1	-	C	T	0.045	0.007	0.000	-0.024	0.023	0.281
	rs17817449	16q12.2	*FTO*	G	T	-0.060	0.006	0.000	0.006	0.016	0.722

Cancer type	SNP	Locus**	Nearest gene*	EA	RA	Beta	SE	p-value	Beta	SE	p-value
							Exposure			Outcome	
	rs1830298	2q33.1	*ALS2CR12*	T	C	-0.056	0.007	0.000	-0.005	0.017	0.770
	rs1895062	9q33.1	*ASTN2-AS1 - ASTN2*	G	A	-0.049	0.006	0.000	0.020	0.016	0.231
	rs2012709	5p13.3	*SUB1*	T	C	0.036	0.006	0.000	0.015	0.016	0.353
	rs204247	6p23	-	A	G	-0.045	0.006	0.000	0.005	0.015	0.731
	rs2223621	6p22.3	*CDKAL1*	C	T	-0.041	0.006	0.000	0.016	0.016	0.319
	rs2236007	14q13.3	*LOC105370455 - PAX9*	A	G	-0.072	0.008	0.000	-0.011	0.020	0.580
	rs2290203	15q26.1	*PRC1-AS1 - PRC1*	A	G	-0.047	0.008	0.000	0.011	0.020	0.589
	rs2432539	16q13	*AMFR*	G	A	-0.035	0.006	0.000	-0.026	0.016	0.115
	rs2594714	19p13.12	-	A	G	-0.043	0.007	0.000	0.009	0.021	0.670
	rs2787486	17q22	*STXBP4*	C	A	-0.076	0.007	0.000	-0.002	0.017	0.890
	rs28512361	22q13.31	*LOC107985535*	A	G	0.061	0.011	0.000	-0.034	0.034	0.327
	rs28539243	16q12.2	*LOC105371274*	A	G	0.049	0.006	0.000	-0.017	0.017	0.310
	rs2943559	8q21.11	*HNF4G*	G	A	0.116	0.011	0.000	0.006	0.028	0.821
	rs2981578	10q26.13	*FGFR2*	T	C	-0.208	0.006	0.000	0.013	0.016	0.435
	rs2992756	1p36.13	*KLHDC7A*	C	T	-0.051	0.006	0.000	-0.019	0.015	0.223
	rs35383942	1q32.1	*PHLDA3*	T	C	0.101	0.014	0.000	0.002	0.039	0.956
	rs3760982	19q13.31	*KCNN4*	G	A	-0.051	0.006	0.000	0.038	0.015	0.013
	rs3817198	11p15.5	*LSP1*	C	T	0.059	0.007	0.000	-0.011	0.018	0.517
	rs3903072	11q13.1	-	T	G	-0.043	0.006	0.000	0.020	0.015	0.206
	rs4233486	1p34.2	-	T	C	0.040	0.007	0.000	0.001	0.017	0.968
	rs4442975	2q35	*LOC101928278*	T	G	-0.127	0.006	0.000	-0.009	0.016	0.579
	rs4496150	16q24.2	*LOC105371393*	A	C	-0.042	0.007	0.000	0.010	0.018	0.575
	rs4562056	5q35.1	-	T	G	0.042	0.007	0.000	0.006	0.017	0.715
	rs4593472	7q32.3	*LINC-PINT*	T	C	-0.044	0.007	0.000	-0.004	0.017	0.809
	rs4784227	16q12.1	*CASC16*	T	C	0.215	0.007	0.000	-0.010	0.018	0.580
	rs4808801	19p13.11	*ELL*	G	A	-0.072	0.007	0.000	0.066	0.016	0.000
	rs4849887	2q14.1	-	C	T	0.095	0.010	0.000	0.004	0.026	0.882

Cancer type	SNP	Locus**	Nearest gene*	EA	RA	Beta	SE	p-value	Beta	SE	p-value
							Exposure			Outcome	
	rs4971059	1q22	*TRIM46*	A	G	0.042	0.006	0.000	-0.008	0.017	0.657
	rs4973768	3p24.1	*SLC4A7*	T	C	0.098	0.006	0.000	0.010	0.016	0.553
	rs514192	8q22.3	*LOC107986961*	T	A	-0.038	0.007	0.000	0.009	0.018	0.606
	rs527616	18q11.2	-	G	C	0.050	0.006	0.000	0.007	0.017	0.685
	rs58058861	3q26.31	*LINC02068*	A	G	0.047	0.007	0.000	0.034	0.020	0.080
	rs58847541	8q24.13	-	A	G	0.063	0.009	0.000	0.044	0.022	0.047
	rs6001930	22q13.1	*MKL1*	C	T	0.120	0.010	0.000	0.002	0.026	0.942
	rs6122906	20q13.13	-	G	A	0.051	0.008	0.000	0.042	0.023	0.061
	rs616488	1p36.22	*PEX14*	G	A	-0.060	0.007	0.000	0.003	0.017	0.841
	rs62355902	5q11.2	-	T	A	0.173	0.008	0.000	-0.037	0.022	0.091
	rs6507583	18q12.3	*SETBP1*	G	A	-0.087	0.012	0.000	0.038	0.032	0.227
	rs6562760	13q22.1	-	G	A	0.044	0.007	0.000	0.020	0.019	0.298
	rs6569648	6q23.1	*L3MBTL3*	T	C	0.051	0.007	0.000	-0.002	0.019	0.898
	rs6596100	5q31.1	*HSPA4*	T	C	-0.044	0.008	0.000	0.041	0.018	0.024
	rs6597981	11p15	*PIDD1*	G	A	0.044	0.006	0.000	0.009	0.016	0.558
	rs6725517	2p23.3	*ADCY3*	G	A	-0.047	0.007	0.000	-0.003	0.016	0.865
	rs676256	9q31.2	*LOC105376214*	T	C	0.098	0.006	0.000	-0.002	0.016	0.927
	rs6796502	3p21.31	-	G	A	0.055	0.006	0.000	-0.004	0.028	0.891
	rs6805189	3p13	*FOXP1*	C	T	-0.034	0.006	0.000	0.002	0.015	0.897
	rs6815814	4p14	-	C	A	0.052	0.007	0.000	0.002	0.018	0.924
	rs6828523	4q34.1	*ADAM29*	A	C	-0.102	0.010	0.000	0.024	0.025	0.335
	rs6882649	5q22.1	*NREP*	T	G	0.039	0.007	0.000	0.011	0.017	0.524
	rs6964587	7q21.2	*AKAP9*	T	G	0.041	0.006	0.000	-0.024	0.016	0.142
	rs704010	10q22.3	*ZMIZ1*	C	T	-0.079	0.006	0.000	0.034	0.016	0.037
	rs7072776	10p12.31	*LOC107984214 - MLLT10*	G	A	-0.062	0.007	0.000	0.029	0.017	0.092
	rs71557345	6p22.2	*LOC102724851*	A	G	-0.081	0.013	0.000	-0.012	0.048	0.809
	rs720475	7q35	*ARHGEF5*	A	G	-0.049	0.007	0.000	0.008	0.017	0.641

Cancer type	SNP	Locus**	Nearest gene*	EA	RA	Beta	SE	p-value	Beta	SE	p-value
							**Exposure**			**Outcome**	
	rs72749841	5q11.1	-	C	T	-0.070	0.011	0.000	-0.085	0.043	0.046
	rs73161324	22q13.2	*XRCC6*	T	C	0.081	0.013	0.000	-0.109	0.048	0.022
	rs745570	17q25.3	-	G	A	-0.039	0.006	0.000	0.027	0.016	0.084
	rs75915166	11q13.3	-	A	C	0.262	0.013	0.000	-0.021	0.036	0.562
	rs7707921	5q14.2	*ATG10*	A	T	0.051	0.007	0.000	-0.002	0.018	0.906
	rs77528541	4q28.1	-	T	G	-0.058	0.010	0.000	0.054	0.032	0.089
	rs78269692	19p13.13	*NFIX*	C	T	0.092	0.015	0.000	0.071	0.051	0.163
	rs7971	7p15.3	*CDCA7L - DNAH11*	G	A	-0.036	0.006	0.000	0.020	0.016	0.211
	rs9397437	6q25	-	A	G	0.184	0.012	0.000	-0.037	0.031	0.228
	rs941764	14q32.11	*CCDC88C*	G	A	0.046	0.006	0.000	-0.021	0.017	0.223
	rs9693444	8p12	-	C	A	-0.063	0.007	0.000	0.002	0.016	0.901
	rs9790517	4q24	*TET2*	T	C	0.048	0.007	0.000	0.016	0.019	0.400
	rs9833888	3p12.1	*CMSS1 - FILIP1L*	T	G	0.046	0.007	0.000	-0.013	0.020	0.506
	rs999737	14q24.1	*RAD51B*	T	C	-0.097	0.007	0.000	0.004	0.019	0.819
Melanoma											
	rs10739221	9q31.2	-	T	C	0.120	0.018	0.000	0.032	0.019	0.081
	rs13016963	2q33-q34	*ALS2CR12*	A	G	0.131	0.021	0.000	0.013	0.016	0.430
	rs1393350	11q14-q21	*LOC107984363 - TYR*	A	G	0.255	0.033	0.000	-0.003	0.018	0.888
	rs1636744	7p21.1	-	T	C	0.091	0.016	0.000	0.003	0.016	0.848
	rs16953002	16q12.2	*FTO*	A	G	0.148	0.021	0.000	-0.044	0.021	0.034
	rs1801516	11q22-q23	*ATM*	A	G	-0.174	0.029	0.000	0.023	0.022	0.290
	rs1805007	16q24.3	*MC1R*	T	C	0.405	0.032	0.000	0.054	0.039	0.158
	rs2284063	22q13.1	*PLA2G6*	G	A	-0.186	0.031	0.000	-0.001	0.016	0.965
	rs2995264	10q24.33	*OBFC1 - STN1*	G	A	0.160	0.027	0.000	-0.035	0.026	0.176
	rs3219090	1q42.12	*PARP1*	T	C	-0.139	0.026	0.000	-0.018	0.017	0.291
	rs35407	5p13.2	*SLC45A2*	A	G	-0.734	0.082	0.000	0.126	0.056	0.024
	rs45430	21q22.3	*MX2*	G	A	-0.128	0.022	0.000	0.009	0.016	0.587

Cancer type	SNP	Locus**	Nearest gene*	EA	RA	Beta	SE	p-value	Beta	SE	p-value
							Exposure			Outcome	
	rs4778138	15q13.1	*OCA2*	G	A	-0.170	0.025	0.000	0.002	0.023	0.939
	rs498136	11q13.3	-	A	C	0.120	0.017	0.000	0.002	0.017	0.897
	rs6001027	22q13.1	*PLA2G6*	G	A	-0.186	0.033	0.000	0.000	0.016	0.998
	rs6059655	20q11.22	*RALY*	A	G	0.322	0.033	0.000	-0.036	0.030	0.231
	rs6750047	2p22.2	*RMDN2*	A	G	0.092	0.016	0.000	0.010	0.016	0.517
	rs6914598	6p22.3	*CDKAL1*	C	T	0.100	0.018	0.000	0.004	0.018	0.808
	rs7412746	1q21.3	*CTXND2 - LOC100996521*	C	T	-0.139	0.021	0.000	0.000	0.016	0.991
Lymphoma											
	rs112998813	13q34	*UPF3A*	C	T	0.329	0.060	0.000	-0.006	0.032	0.857
	rs116446171	6p25.3	-	G	C	0.788	0.083	0.000	0.021	0.058	0.721
	rs12289961	11q12.1	-	T	C	0.255	0.046	0.000	-0.006	0.020	0.748
	rs13255292	8q24.21	*PVT1*	T	C	0.199	0.028	0.000	-0.021	0.017	0.218
	rs1432295	2p16.1	-	G	A	0.199	0.035	0.000	-0.032	0.016	0.043
	rs17749561	18q21.33	-	G	A	0.293	0.048	0.000	-0.030	0.026	0.263
	rs2019960	8q24.21	-	G	A	0.285	0.038	0.000	-0.009	0.019	0.631
	rs34972832	16p13.3	*CLEC16A*	A	G	0.215	0.038	0.000	-0.062	0.019	0.001
	rs3781093	10p14	*GATA3*	T	C	0.329	0.046	0.000	0.012	0.022	0.587
	rs3806624	3p24.1	*EOMES*	G	A	0.231	0.032	0.000	-0.001	0.016	0.946
	rs4459895	3q28	*LPP*	A	C	0.329	0.040	0.000	0.065	0.022	0.004
	rs4937362	11q24.3	*LOC105369568*	T	C	0.174	0.027	0.000	0.009	0.017	0.573
	rs4938573	11q23.3	-	C	T	0.293	0.032	0.000	0.043	0.021	0.035
	rs6928977	6q23.3	*AHI1*	G	T	0.207	0.031	0.000	0.011	0.016	0.487
	rs79480871	2p23.3	-	T	C	0.293	0.053	0.000	0.027	0.034	0.421
	rs9482849	6q22.33	-	C	T	0.182	0.032	0.000	0.017	0.022	0.442
	rs9831894	3q13.33	*CD86*	C	A	-0.186	0.032	0.000	-0.015	0.016	0.355
Ovarian cancer											

Cancer type	SNP	Locus**	Nearest gene*	EA	RA	Beta	SE	p-value	Beta	SE	p-value
							**Exposure**			**Outcome**	
	rs10088218	8q24.21	*LINC00824*	A	G	-0.174	0.029	0.000	-0.004	0.023	0.848
	rs11782652	8q21.13	*CHMP4C*	G	A	0.215	0.035	0.000	0.061	0.031	0.051
	rs1243180	10p12	*MLLT10*	A	T	0.095	0.017	0.000	-0.031	0.017	0.060
	rs13113999	4q32.3	-	T	G	0.207	0.038	0.000	-0.003	0.018	0.887
	rs150293538	8q21.11	*LINC01111*	T	C	0.784	0.131	0.000	-0.006	0.062	0.918
	rs2072590	2q31	*HAGLR - HAGLROS*	T	G	0.148	0.020	0.000	0.040	0.017	0.016
	rs2363956	19p13.11	*ANKLE1*	T	G	0.148	0.022	0.000	0.014	0.016	0.397
	rs2665390	3q25	*LOC107986148 - TIPARP*	C	T	0.215	0.040	0.000	0.002	0.030	0.942
	rs320203	9q31.1	-	A	C	0.255	0.045	0.000	-0.006	0.022	0.774
	rs3814113	9p22.2	-	C	T	-0.261	0.028	0.000	0.014	0.017	0.405
	rs4803217		*IFNL3*	A	C	-0.400	0.056	0.000	-0.007	0.019	0.693
	rs6005807	22q12.1	*LOC101929594 - TTC28*	C	T	0.157	0.027	0.000	-0.022	0.028	0.427
	rs635634	9q34.2	-	T	C	0.113	0.020	0.000	-0.006	0.020	0.779
	rs752590	2q13	-	G	A	0.293	0.053	0.000	0.013	0.019	0.488
	rs757210	17q12	*HNF1B*	A	G	0.113	0.018	0.000	0.009	0.017	0.601
	rs7902587	10q24.33	-	T	C	0.255	0.046	0.000	-0.052	0.028	0.057
	rs7953249	12q24.31	-	G	A	0.077	0.013	0.000	-0.022	0.016	0.157
	rs8098244	18q11.2	*LAMA3*	A	G	0.174	0.032	0.000	0.025	0.017	0.146
	rs9870207	3q28	-	G	A	0.174	0.032	0.000	-0.012	0.017	0.482

Supplementary Table 7. Literature-retrieved genetic variants missing in IGAP and their corresponding proxy variants, where available. Seven of the 26 missing SNPs were replaced with proxies (r^2>0.9 in the European subpopulation of the 1000 Genomes Project**).** Proxies**,** EAF, R^2, and D' values were retrieved from the National Cancer Institute LDlink platform. Proxy SNP effect alleles were assigned according to correlation information between alleles, provided on the LDpair tool on the LDlink platform. EA= effect allele; RA= reference (non-effect) allele; EAF= effect allele frequency.

Cancer type	Cancer- associated SNP				International Genomics of Alzheimer's Project proxy					
	SNPid	EA	RA	EAF	SNPid	EA	RA	EAF	R^2	D'
Pancreatic cancer										
	rs35226131	T	C	0.01	-	-	-	-	-	-
Upper aerodigestive tract cancer										
	rs3828805	C	T	0.82	rs9274626	C	T	0.82	0.94	0.97
Lung cancer										
	rs11571833	T	A	0.00	-	-	-	-	-	-
	rs1787996	G	A	1.00	-	-	-	-	-	-
	rs77468143	G	T	0.19	rs146782107	C	T	0.19	0.95	1
Prosate cancer										
	rs3096702	A	G	0.30	-	-	-	-	-	-
	rs6062509	C	A	0.29	rs1056441	T	C	0.28	0.99	1
	rs6530332	A	G	0.22	-	-	-	-	-	-
	rs188140481	A	T	0.00	-	-	-	-	-	-
	rs138213197	T	C	0.00	-	-	-	-	-	-
	rs5945572	A	G	0.24	-	-	-	-	-	-
Leukemia										
	rs71597109	C	T	0.78	rs17200824	A	G	0.74	0.99	1
	rs9880772	A	G	0.70	rs1353286	G	T	0.72	1	1
	rs10069690	T	C	0.35	-	-	-	-	-	-
	rs2456449	G	A	0.31	rs897586	A	G	0.33	0.99	1
Breast cancer										
	rs11571833	T	A	0.00	-	-	-	-	-	-
	rs11076805	A	C	0.19	-	-	-	-	-	-
	rs72826962	T	C	0.00	-	-	-	-	-	-
	rs17879961	G	A	0.00	-	-	-	-	-	-
	rs132390	T	C	0.97	-	-	-	-	-	-
Melanoma										
	rs62389423	A	G	0.03	-	-	-	-	-	-
	rs139996880	A	G	0.14	-	-	-	-	-	-
	rs187843643	T	C	0.00	-	-	-	-	-	-
	rs1204552	A	T	0.00	-	-	-	-	-	-
Lymphoma										

Cancer type	Cancer- associated SNP				International Genomics of Alzheimer's Project proxy					
	**SNPid**	**EA**	**RA**	**EAF**	**SNPid**	**EA**	**RA**	**EAF**	**R^2**	**D'**
	rs2523607	A	T	0.06	-	-	-	-	-	-
	rs12195582	T	C	0.42	-	-	-	-	-	-
Ovarian cancer										
	rs688187	A	G	0.3	rs4803217	A	C	0.35	0.97	1

Appendix 1.

Box A: Preparing exposure datafile for Mendelian randomization

The following set of information was first extracted from the GWAS literature (In the order shown) for the cancer type under study and saved as a .txt file in the working directory:

(1) SNP (snp id) (2) effect_allele (3) other_allele (4) OR (odds ratio) (5) se (standard error) (6) pval (p-value)

```
> setwd(“path to working directory")
> Exposure = read.table(file.choose(), header=TRUE, sep="\t")
> Exposure[paste("beta")] <- log(Exposure[4])
> Exposure$OR<- NULL
> Exposure [paste("pdivtwo")] <- (Exposure[5]/2)
> Exposure [paste("z")] <- -qnorm (Exposure $pdivtwo)
> Exposure [paste("se")] <- Exposure [6]/ LCSNPs [8])
> Exposure [paste("se")] <- abs(Exposure $se)
> Exposure$pdivtwo<- NULL
> Exposure$z<- NULL
> View(Exposure)
> write.table(Exposure, "Exposure_clean.txt", sep="\t",
row.names=FALSE)
```

Box B: Preparing outcome datafile for Mendelian randomization

The pre-cleaned IGAP dataset was retrieved from: http://web.pasteur-lille.fr/en/recherche/u744/igap/igap_download.php

```
> setwd("path to working directory")
> Outcome = read.table(file.choose(), header=TRUE, sep="\t")
> names(Outcome)[names(Outcome)=="MarkerName"] <- "SNP"
> names(Outcome)[names(Outcome)=="Effect_allele"] <-
"effect_allele"
> names(Outcome)[names(Outcome)=="Non_Effect_allele"] <-
"other_allele"
> names(Outcome)[names(Outcome)=="Beta"] <- "beta"
> names(Outcome)[names(Outcome)=="SE"] <- "se"
> names(Outcome)[names(Outcome)=="Pvalue"] <- "pval"
> df1$Chromosome <- NULL
> df1$Position <- NULL
> write.table(Outcome, "Outcome_clean.txt", sep="\t ",
row.names=FALSE)
```

Box C: Running the Mendelian randomization analysis

```
> setwd("path to working directory")
> library(TwoSampleMR)
> Exposure= read.table(file.choose(), header=TRUE, sep="\t")
> cancer_exp_dat <- format_data(Exposure)
> outcome_dat <- read_outcome_data(snps =
cancer_exp_dat$SNP, filename="outcome_clean.txt", sep= "\t")
> dat <- harmonise_data(exposure_dat = gcancer_exp_dat,
outcome_dat, action=1)
> res <- mr(dat, method_list=c("mr_ivw"))
> res
##Forest plot
> res_single <- mr_singlesnp(dat, all_method=c("mr_ivw"))
> p2 <- mr_forest_plot(res_single, exponentiate=TRUE)
> p2[[1]]
##Scatter plot
> p1 <- mr_scatter_plot(res, dat)
➢ p1[[1]]
```

Sensitivity Analyses:

```
##MR-Egger intercept test
> mr_pleiotropy_test (dat)
##Leave-one-out test
> res_loo <- mr_leaveoneout(dat)
> p3 <- mr_leaveoneout_plot(res_loo)
> p3[[1]]
##Funnel plot
> res_single <- mr_singlesnp(dat, all_method=c("mr_ivw"))
> p4 <- mr_funnel_plot(res_single)
> p4[[1]]
```

Box D: Calculating Bayes False Discovery Rate

```
> library(gap)
> library(tidyverse)
> T <- 0.8
> pi0 <- c(0.5, 0.1, 0.01, 0.001)
> ORhi <- 0.8
> W <- (log(ORhi)/1.96)^2
> logOR <- -0.0534
> selogOR <- 0.01774
> r <- W/(W+selogOR^2)
> z <- logOR/selogOR
> r <- W/(W+selogOR^2)
> ABF <- exp(-z^2*r/2)/sqrt(1-r)
> FF <- (1-pi0)/pi0
> BFDPex <- FF*ABF/(FF*ABF+1)
> BFDPex
```

Box E: Generating a forest plot to depict inverse-variance weighted effect estimates for all cancer types in study

The following set of information was first saved as a .txt file in the working directory:

(1) Cancertype (cancer site) (2) or (odds ratio) (3) lcl (lower confidence limit) (4) ucl (upper confidence limit) (5) cancer (category, e.g. smoking-related cancer) (6) order (the relative order in which each cancer type should appear on plot)

```
> setwd("path to working directory")
> dat = read.table(file.choose(), header=TRUE, sep="\t")
> ggplot(dat, aes(x = reorder (cancertype, -order), y = or, color =
cancer)) +
  geom_pointrange(aes(ymin = lcl, ymax = ucl), shape = 16) +
  scale_colour_manual(values = c("lightsteelblue3", " coral1",
"midnightblue")) +
  scale_y_log10(breaks = c(0.80, 0.85, 0.90, 0.95, 1.0, 1.05, 1.1, 1.15,
1.2, 1.5, 2.0)) +
  geom_hline(yintercept = 1) +
  coord_flip() +
  theme_bw() +
  theme(legend.position="right",
        axis.text.x=element_text(size = 12),
        axis.text.y=element_text(size = 12),
        axis.title.x = element_text(size=15),
        axis.title.y = element_text(size=15)) +
  labs(title = "Mendelian Randomization Effect Estimates", x =
"Cancer type", y = "Odds ratio", colour = "Cancer")
```

Appendix 2.

Figure A. Leave-one-out sensitivity analysis for lung cancer. Each blue circle represents the inverse-variance weighted Mendelian randomization estimate for the causal effect of lung cancer on Alzheimer's disease while excluding the corresponding genetic variant from analysis. The orange circle represents the inverse-variance weighted Mendelian randomization estimate for the causal effect of lung cancer on Alzheimer's disease using all SNPs. While two SNPs had a marginal influence on the overall estimate for the association between lung cancer and Alzheimer's disease by skewing results away from statistical significance, the exclusion of no particular SNP led to substantial changes in the overall estimate.

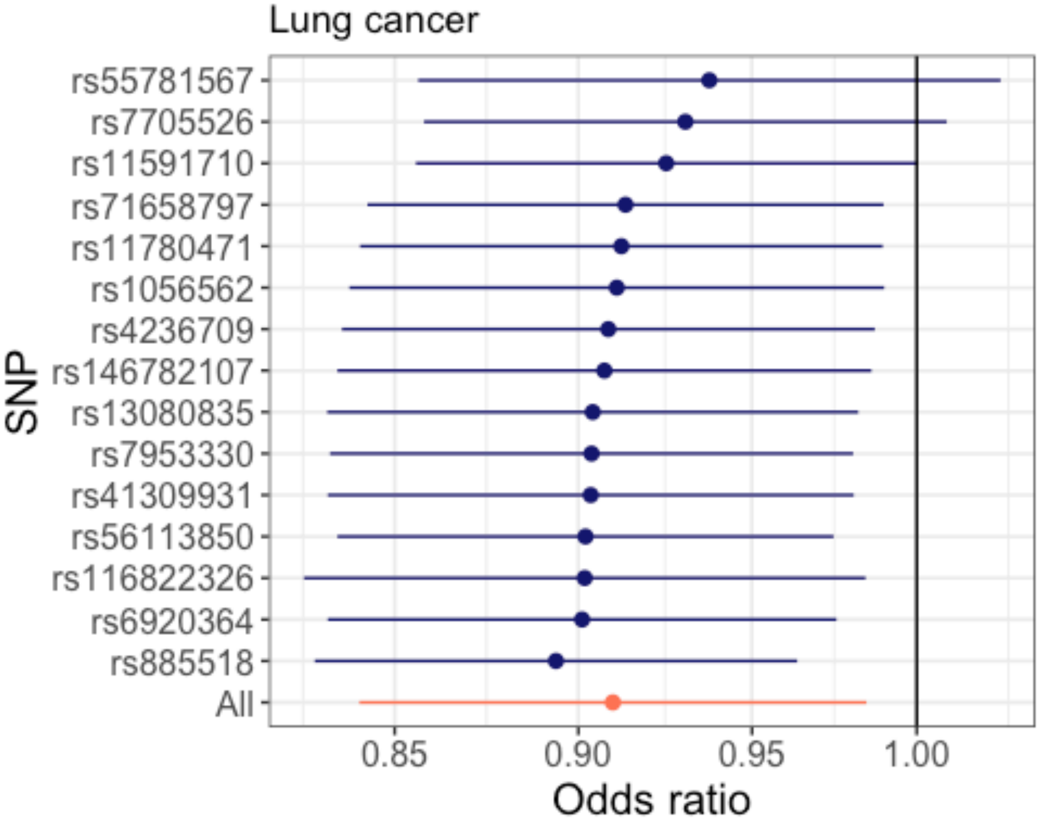

Figure B. Leave-one-out sensitivity analysis for leukemia. Each blue circle represents the inverse-variance weighted Mendelian randomization estimate for the causal effect of leukemia on Alzheimer's disease while excluding the corresponding genetic variant from analysis. The orange circle represents the inverse-variance weighted Mendelian randomization estimate for the causal effect of leukemia on Alzheimer's disease using all SNPs. The exclusion of no particular SNP led to substantial changes in the overall estimate.

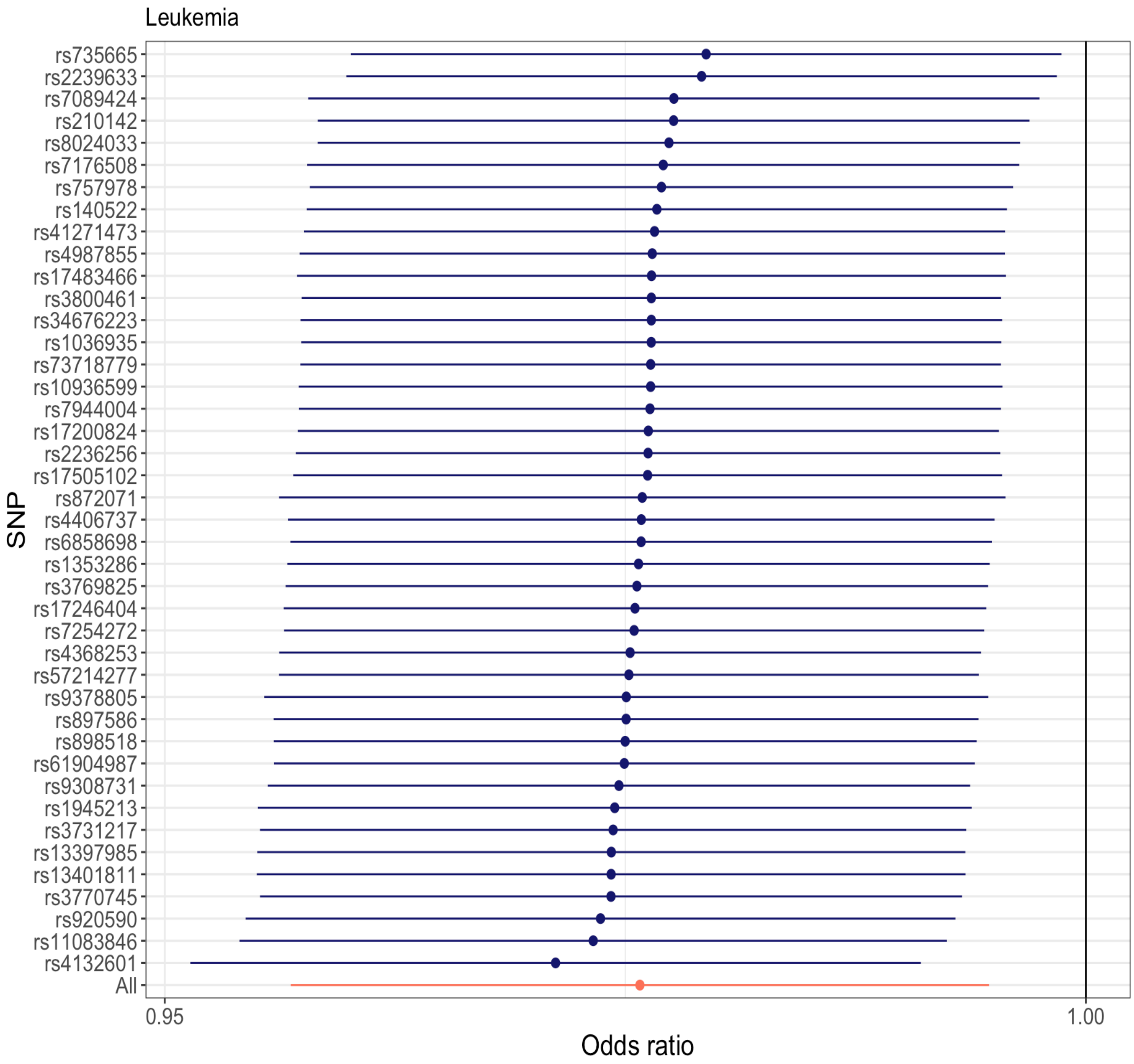

Figure C. Leave-one-out sensitivity analysis for breast cancer. Each blue circle represents the inverse-variance weighted Mendelian randomization estimate for the causal effect of breast cancer on Alzheimer's disease while excluding the corresponding genetic variant from analysis. The orange circle represents the inverse-variance weighted Mendelian randomization estimate for the causal effect of breast cancer on Alzheimer's disease using all SNPs. While two SNPs had a marginal influence on the overall estimate for the association between breast cancer and

Alzheimer’s disease by driving results away from statistical significance, the exclusion of no particular SNP led to substantial changes in the overall estimate.

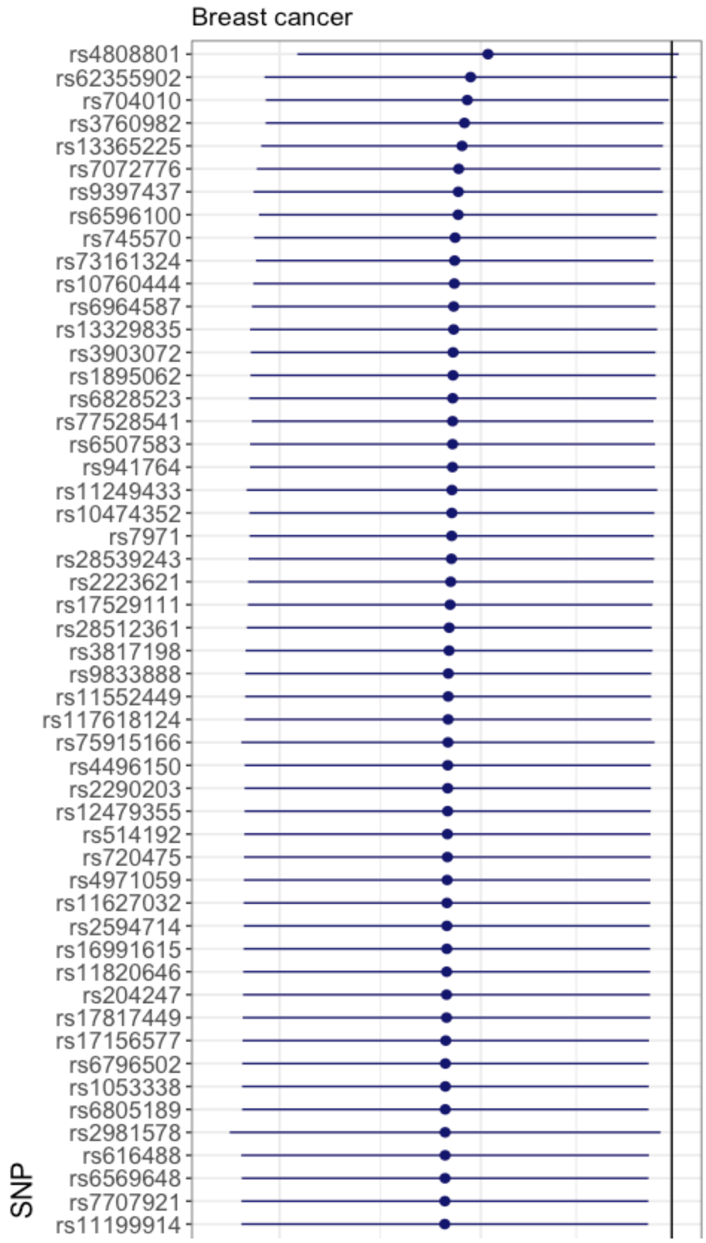

Continued on next page

FIGURE 40 *continued*

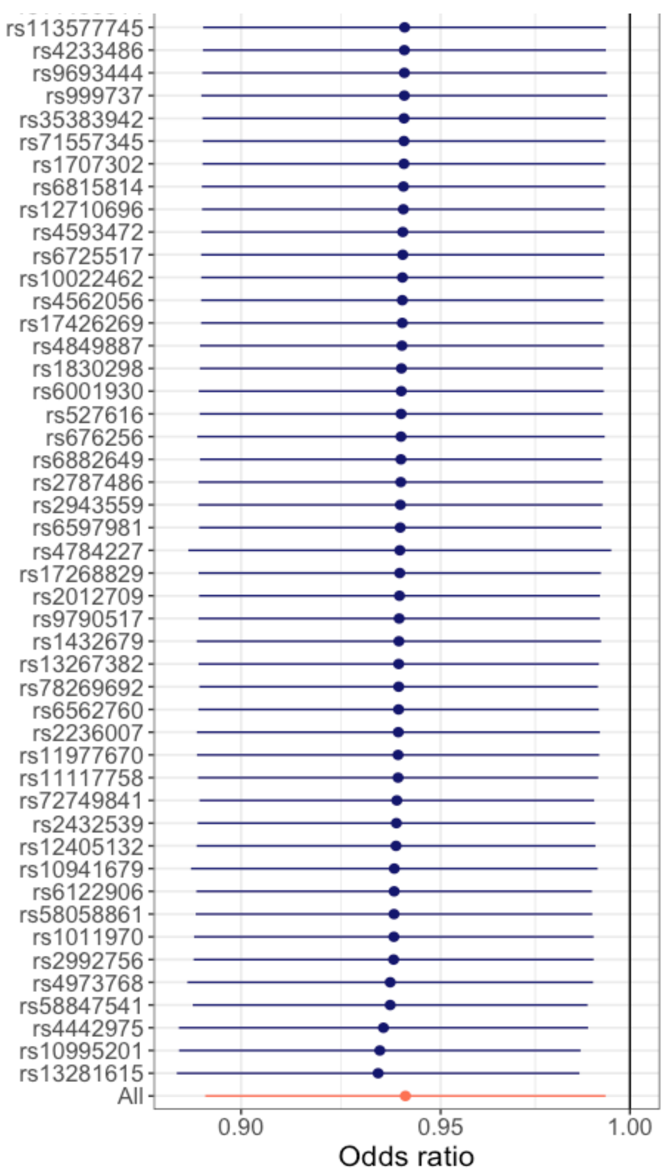

Figure D. Leave-one-out sensitivity analysis for smoking-related cancers. Each blue circle represents the inverse-variance weighted Mendelian randomization estimate for the causal effect of non-smoking related cancers on Alzheimer's disease while excluding the corresponding genetic variant from analysis. The orange circle represents the inverse-variance weighted Mendelian randomization estimate for the causal effect of non-smoking related cancers on Alzheimer's disease using all SNPs. The exclusion of no particular SNP led to substantial changes in the overall estimate.

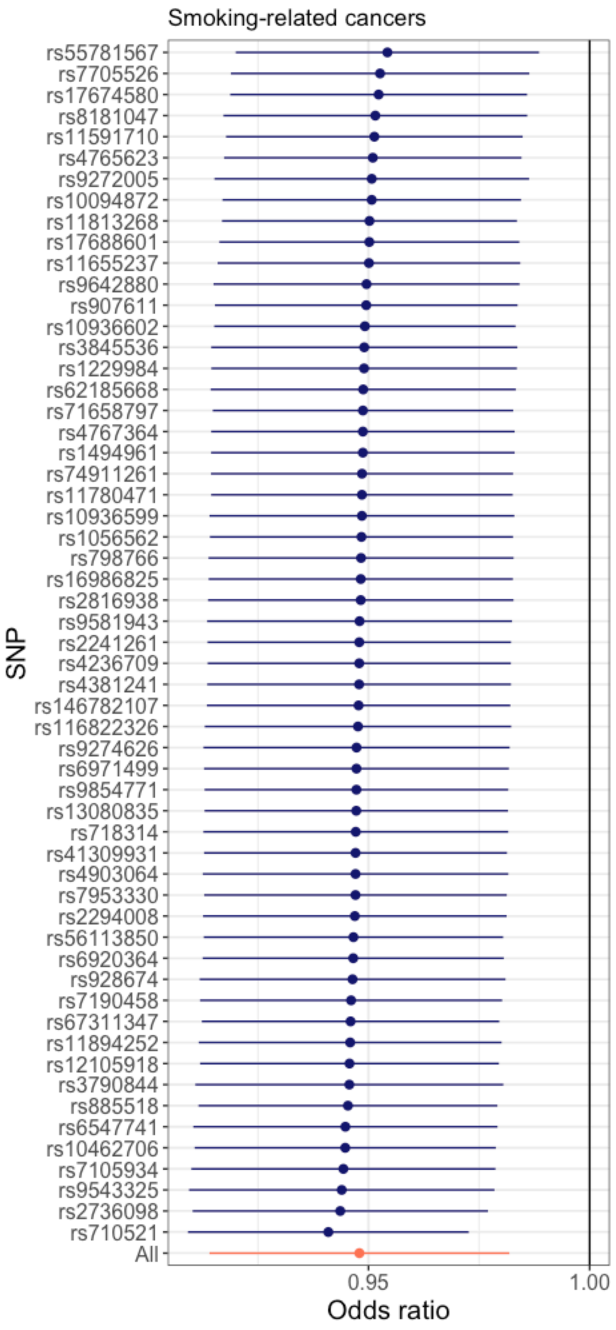

Figure E. Leave-one-out sensitivity analysis for non-smoking related cancers. Each blue circle represents the inverse-variance weighted Mendelian randomization estimate for the causal effect of non-smoking related cancers on Alzheimer's disease while excluding the corresponding genetic variant from analysis. The orange circle represents the inverse-variance weighted Mendelian randomization estimate for the causal effect of non-smoking related

cancers on Alzheimer's disease using all SNPs. The exclusion of no particular SNP led to substantial changes in the overall estimate.

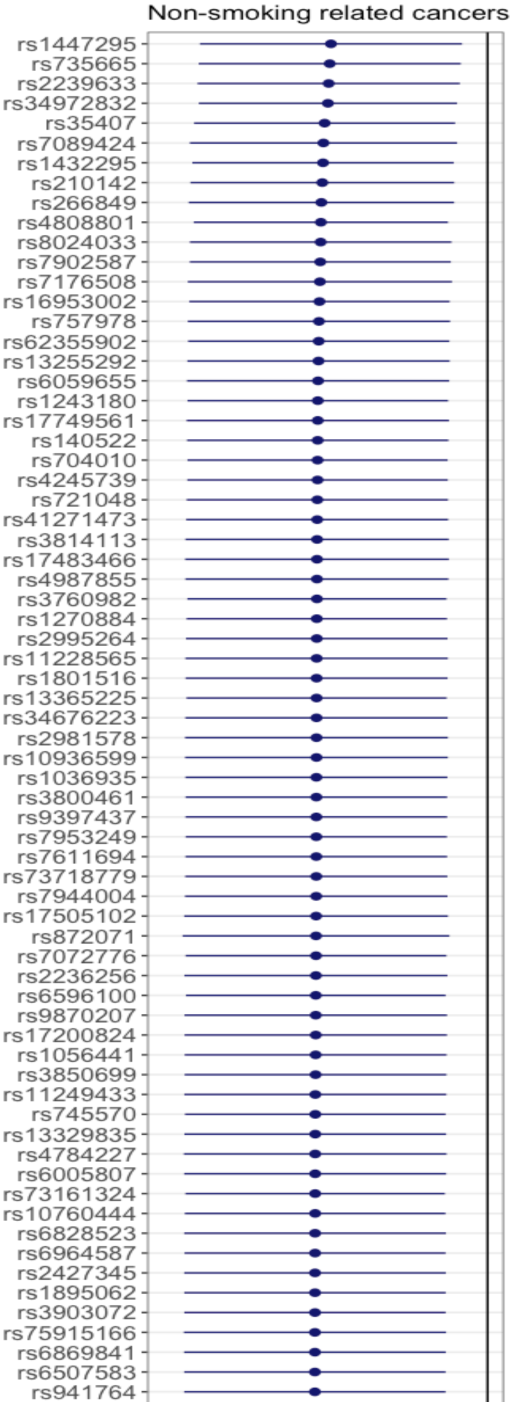

Continued on next page

FIGURE 42 *continued*

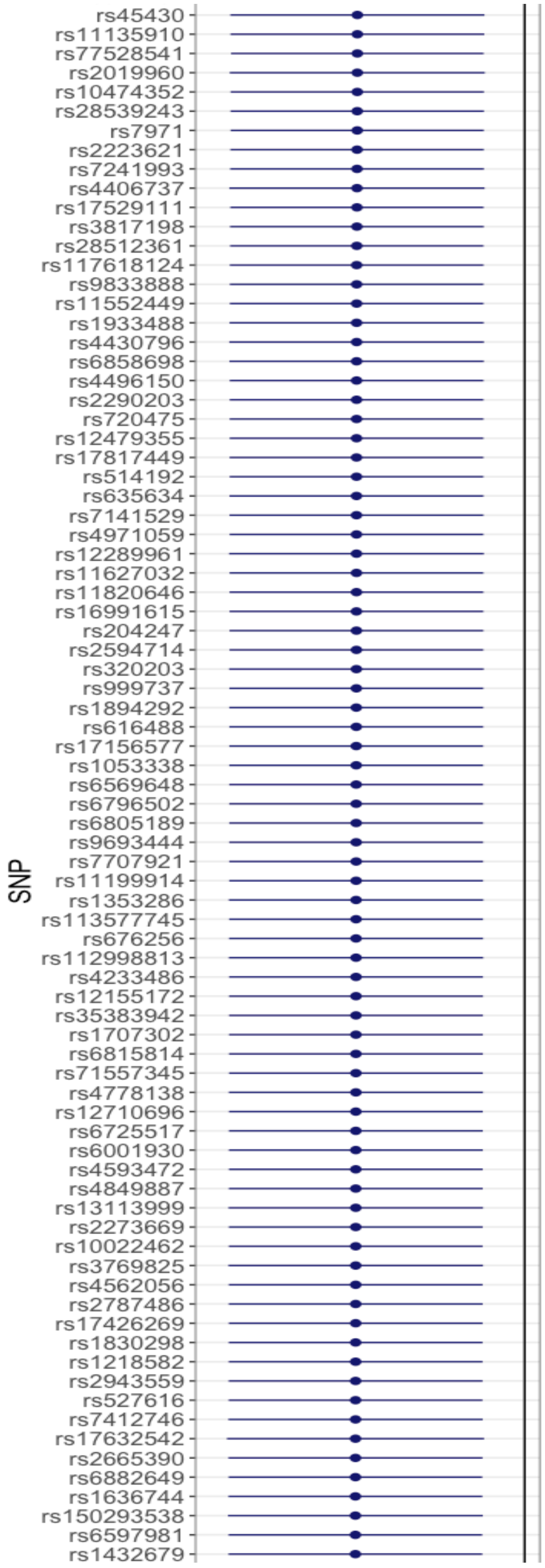

Continued on next page

FIGURE 42 *continued*

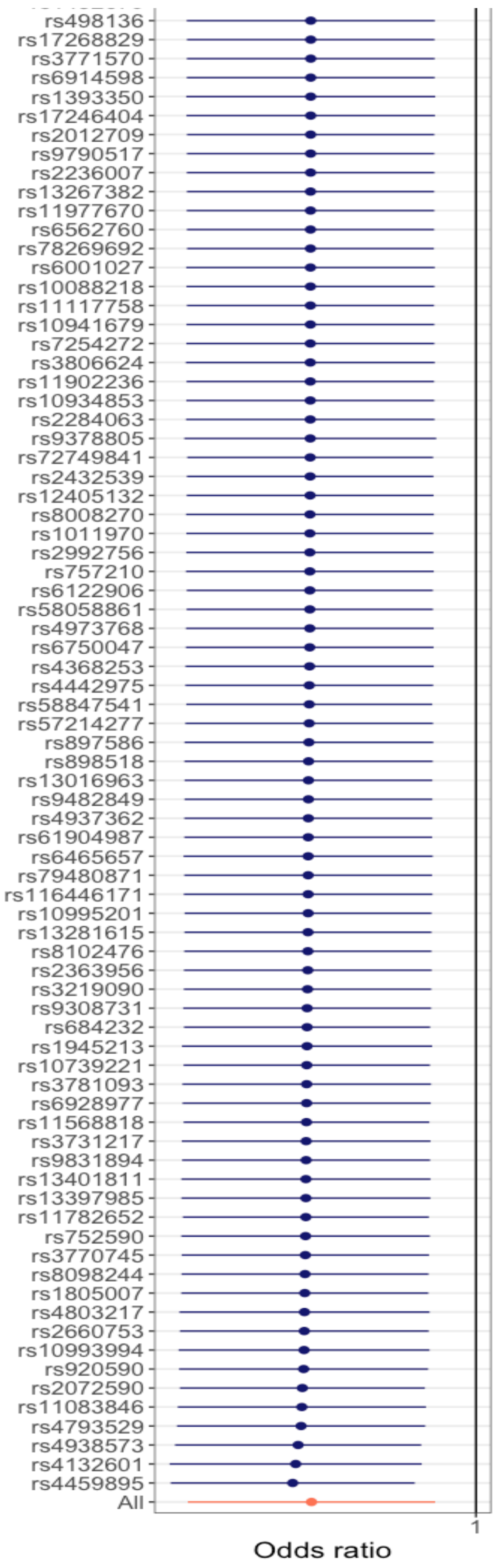
rs498136
rs17268829
rs3771570
rs6914598
rs1393350
rs17246404
rs2012709
rs9790517
rs2236007
rs13267382
rs11977670
rs6562760
rs78269692
rs6001027
rs10088218
rs11117758
rs10941679
rs7254272
rs3806624
rs11902236
rs10934853
rs2284063
rs9378805
rs72749841
rs2432539
rs12405132
rs8008270
rs1011970
rs2992756
rs757210
rs6122906
rs58058861
rs4973768
rs6750047
rs4368253
rs4442975
rs58847541
rs57214277
rs897586
rs898518
rs13016963
rs9482849
rs4937362
rs61904987
rs6465657
rs79480871
rs116446171
rs10995201
rs13281615
rs8102476
rs2363956
rs3219090
rs9308731
rs684232
rs1945213
rs10739221
rs3781093
rs6928977
rs11568818
rs3731217
rs9831894
rs13401811
rs13397985
rs11782652
rs752590
rs3770745
rs8098244
rs1805007
rs4803217
rs2660753
rs10993994
rs920590
rs2072590
rs11083846
rs4793529
rs4938573
rs4132601
rs4459895
All
1
Odds ratio

Figure F. Leave-one-out sensitivity analysis for all cancers. Each blue circle represents the inverse-variance weighted Mendelian randomization estimate for the causal effect of non-smoking related cancers on Alzheimer's disease while excluding the corresponding genetic variant from analysis. The orange circle represents the inverse-variance weighted Mendelian randomization estimate for the causal effect of non-smoking related cancers on Alzheimer's disease using all SNPs. The exclusion of no particular SNP led to substantial changes in the overall estimate.

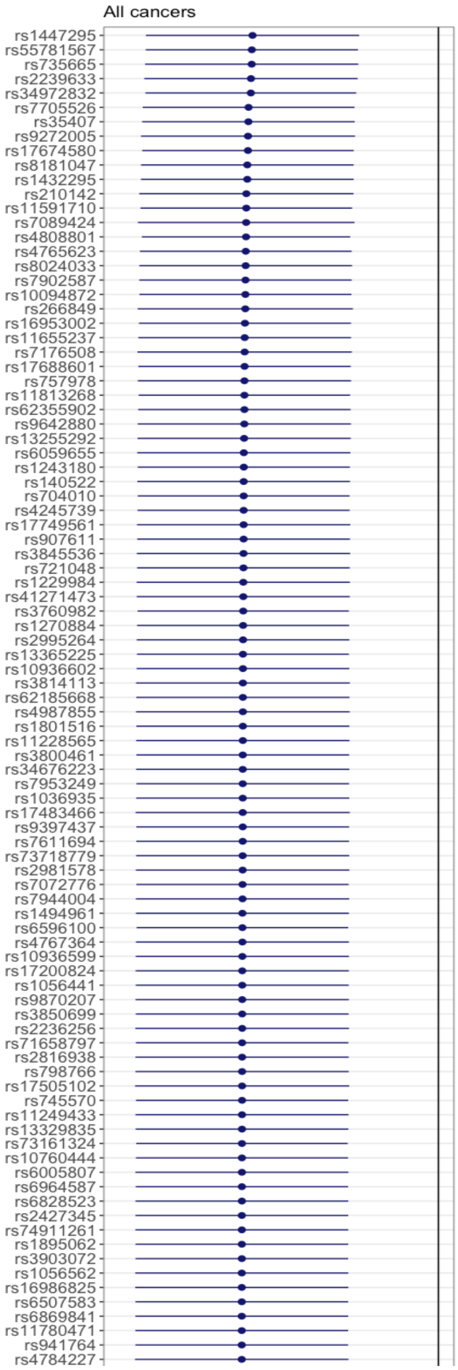

Continued on next page

***FIGURE 43** continued*

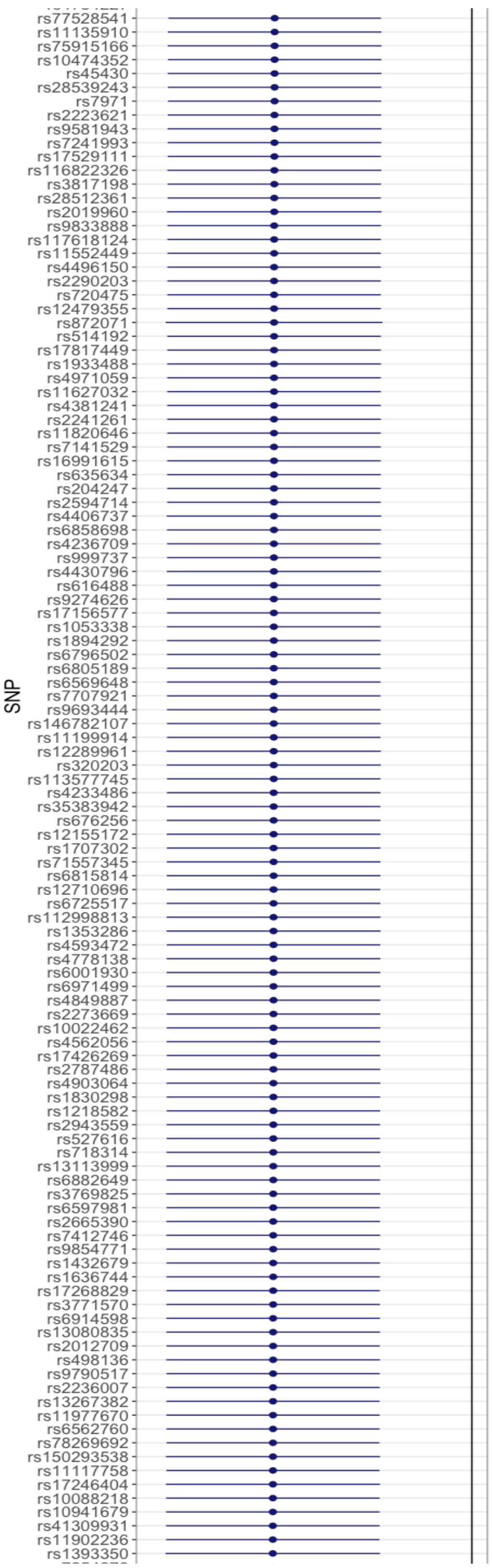

Continued on next page

FIGURE 43 continued

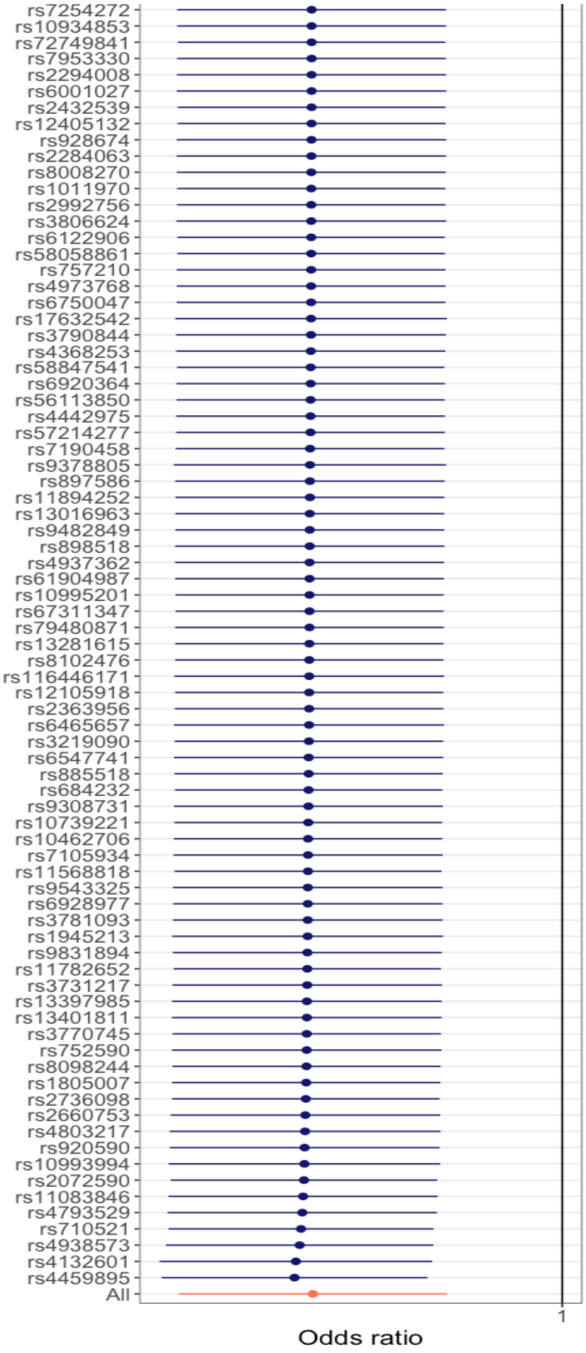

References

1. Beach TG, Monsell SE, Phillips LE, et al. Accuracy of the clinical diagnosis of Alzheimer disease at National Institute on Aging Alzheimer Disease Centers, 2005–2010. *Journal of neuropathology and experimental neurology* 2012;71(4):266-73.
2. McKhann G, Drachman D, Folstein M, et al. Clinical diagnosis of Alzheimer's disease Report of the NINCDS-ADRDA Work Group* under the auspices of Department of Health and Human Services Task Force on Alzheimer's Disease. *Neurology* 1984;34(7):939-39.
3. Braak H, Braak E. Neuropathological stageing of Alzheimer-related changes. *Acta neuropathologica* 1991;82(4):239-59.
4. Mirra SS, Heyman A, McKeel D, et al. The Consortium to Establish a Registry for Alzheimer's Disease (CERAD) Part II. Standardization of the neuropathologic assessment of Alzheimer's disease. *Neurology* 1991;41(4):479-79.
